# Long-Term Outcome of Spontaneous Isolated Dissection of the Superior Mesenteric Artery

**DOI:** 10.3400/avd.oa.25-00081

**Published:** 2026-02-18

**Authors:** Zaiqiang Yu, Norihiro Kondo, Yoshiaki Saito, Kazuyuki Daitoku, Ikuo Fukuda, Masahito Minakawa

**Affiliations:** Department of Thoracic and Cardiovascular Surgery, Hirosaki University Graduate School of Medicine, Hirosaki, Aomori, Japan

**Keywords:** acute spontaneous isolated dissection of the superior mesenteric artery (SIDSMA), controversial treatment, open surgery, long-term outcome

## Abstract

**Objectives:**

We aimed to elucidate the long-term outcomes of acute symptomatic spontaneous isolated dissection of the superior mesenteric artery (SIDSMA) to inform optimal decision-making during the acute phase.

**Methods:**

We retrospectively collected and analyzed data from 14 consecutive patients diagnosed with SIDSMA by using computed tomography angiography (CTA) between January 2010 and August 2024.

**Results:**

The cohort comprised 13 males and 1 female, with a mean age of 59.36 ± 14.90 years. All patients presented with acute abdominal pain, and some experienced vomiting. Thirteen patients received conservative treatment, while only 1 patient underwent open surgery with extra-anatomical bypass; this patient required no further intervention 10 years postoperatively. One of the patients, whose abdominal pain worsened with food intake, showed SMA stenosis and decreased intestinal blood flow. His symptoms improved after heparin anticoagulation therapy followed by direct oral anticoagulant therapy. Over a follow-up period of 7.20 ± 3.21 years, none of the patients experienced recurrent SIDSMA-related abdominal pain, and all survived without the need for additional invasive treatment.

**Conclusions:**

Conservative treatment effectively manages SIDSMA over the long term without reintervention. Early diagnosis and management of intestinal ischemia are essential for optimal treatment outcomes.

## Introduction

Acute spontaneous isolated dissection of the superior mesenteric artery (SIDSMA) is increasingly reported in recent literature.^1,2)^ Several treatment options are available, including conservative treatment,^3)^ open surgery,^4)^ and endovascular surgery^5)^; however, comprehensive long-term outcome data remain limited. Our previous report detailed 10 cases of SIDSMA diagnosed and treated at our hospital, with follow-up conducted at a related facility.^4)^ Although conservative treatment is primarily used to treat SIDSMA, endovascular interventions are becoming increasingly common in acute cases.^5)^ Given the potential for changes in the dissection status over time, it is possible that, analogous to preemptive thoracic endovascular aortic repair (TEVAR) for Stanford type B uncomplicated acute aortic dissection, the treatment strategy for SIDSMA might evolve based on long-term outcomes.^6)^ We aimed to clarify and elucidate the decision-making processes for acute symptomatic SIDSMA based on our single-center experience and extended follow-up.

## Materials and Methods

From January 2010 to August 2024, 14 patients were diagnosed with acute symptomatic SIDSMA using contrast-enhanced computed tomography (CT) angiography, with informed consent obtained at Hirosaki University Hospital. The treatment decisions were based on the presence of peritoneal irritation, signs of intestinal ischemia, or persistent symptoms.^4)^ Conservative treatment consisted of pain management, bowel rest, and hypertension control, typically with a hospital stay of up to 2 weeks,^7)^ and included anticoagulation therapy to prevent thromboembolism. Data from these in-hospital treatments were retrospectively collected and analyzed. Follow-up was scheduled for each patient with contrast-enhanced CT at 3 months, 6 months, and 1 year after onset, and annually thereafter, conducted at their local clinic. Both clinical and CT data were collected for long-term analysis.

## Results

This study included 13 male and 1 female participant; the mean age was 54.3 ± 14.1 years. All the patients presented with acute abdominal pain (**Table 1**). Three patients exhibited symptoms indicative of possible intestinal ischemia, including 1 case of peritonitis. The details of these cases are as follows.

**Table 1:** Patient information

No.	Onset age	Sakamoto type	Yun type	Yu-Kondo type	Angulation between SMA and distal aorta	Follow-up (years)	Change of dissection
1	78	I	I	IIa	>70°	11.1	None
2	49	III	IIb	Ia	<70°	11	None
3	75	III	IIb	Ia	<70°	9.4	None
4	52	I	I	IIa	<70°	9	None
5	75	IV	IIb	Ib	<70°	8.8	None
6	60	III	IIb	Ia	<70°	8	Enlargement
7	47	II	IIa	IIb	<70°	8	Disappearance
8	44	IV	IIb	Ia	<70°	8	Disappearance
9	76	IV	IIb	Ia	<70°	7.8	None
10	60	III	IIb	Ib	<70°	7.7	None
11	82	IV	IIb	Ib	>70°	6.7	Disappearance
12	42	III	IIb	Ia	>70°	3.1	Disappearance
13	49	III	IIb	Ib	>70°	1.1	None
14	42	IV	IIb	Ia	<70°	1.1	None

SMA: superior mesenteric artery

**Fig. 1:**
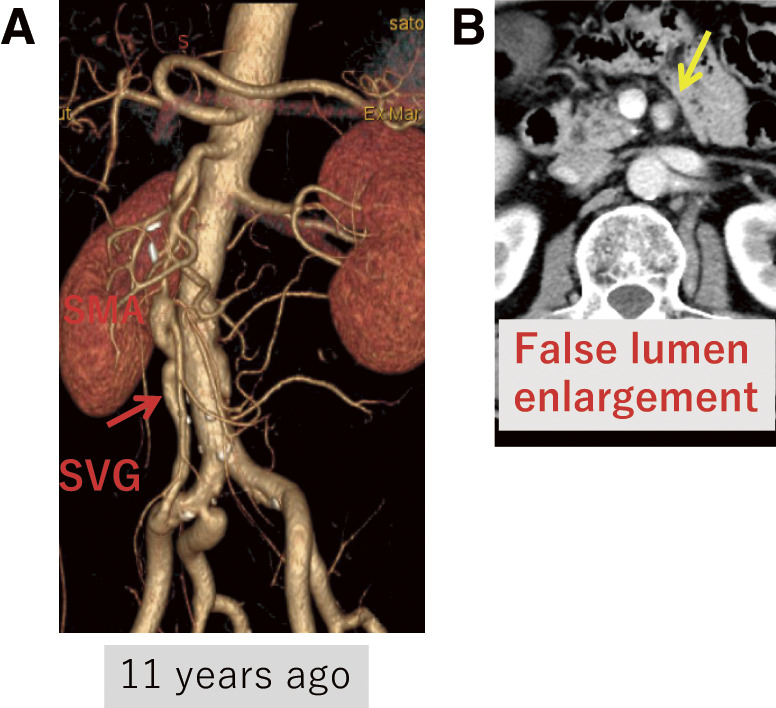
Patient no. 6. (**A**) Rt EIA-SVG-SMA bypass (red arrow). (**B**) False lumen enlargement without additional treatment (yellow arrow). EIA: external iliac artery; Rt: right; SMA: superior mesenteric artery; SVG: saphenous vein graft

Case 1: A 60-year-old male presented with acute abdominal pain and vomiting. The patient experienced morphine-resistant pain. Computed tomography angiography (CTA) revealed occlusion of the superior mesenteric artery (SMA) trunk, and intestinal ischemia was suspected. An extra-anatomical bypass was performed from the left external iliac artery (EIA) to the SMA using the great saphenous vein. Follow-up CTA conducted 10 years postoperatively showed that the bypass graft remained patent without signs of graft degeneration. The maximum diameter of the SMA and the false lumen had increased from 5.0 to 6.7 mm and from 6.0 to 8.0 mm, respectively, over the decade (**Fig. 1**). No therapeutic intervention was required for this enlargement.

**Fig. 2:**
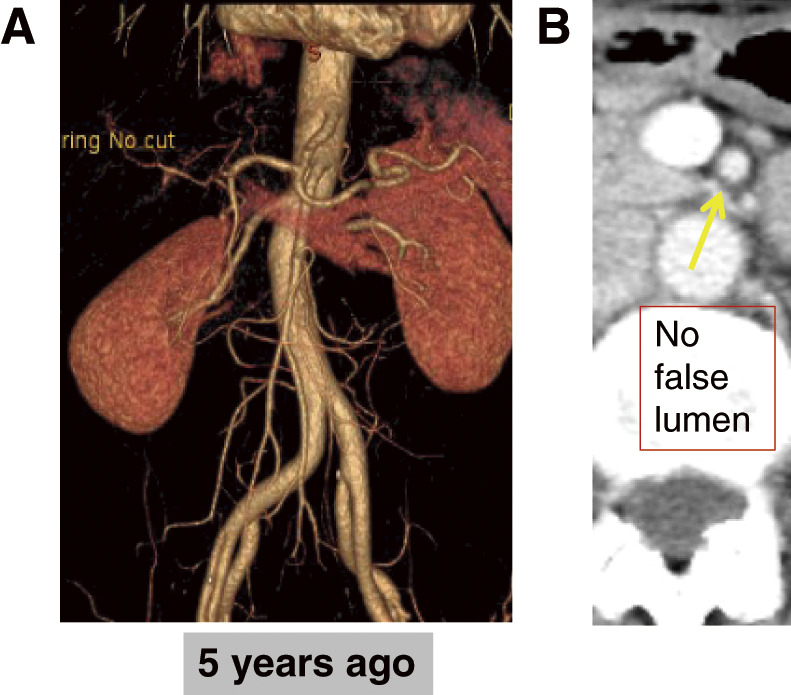
Patient no. 7. (**A**) Angiography showed no reduction in bowel blood flow. (**B**) Disappearance of the false lumen in the SMA (yellow arrow). SMA: superior mesenteric artery

Case 2: A 47-year-old female with acute abdominal pain and vomiting was suspected of having intestinal ischemia. Urgent angiography revealed no intestinal malperfusion, and the false lumen disappeared after 3 years (**Fig. 2**).

**Fig. 3:**
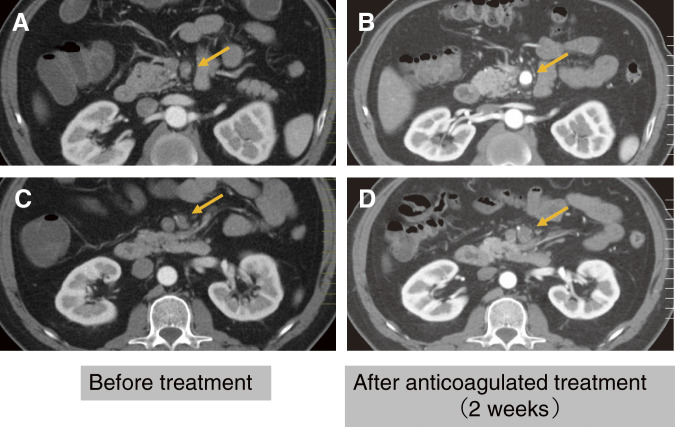
Patient no. 13. (**A**, **C**) Stenosis of the SMA trunk induced by thrombosis in the false lumen (golden arrow). (**B**, **D**) Improved SMA perfusion following anticoagulant therapy after 7 days (golden arrow). SMA: superior mesenteric artery

Case 3: A 49-year-old male was admitted for conservative treatment of SIDSMA. The patient experienced abdominal angina exacerbated by eating. CTA indicated true lumen stenosis compressed by a thrombosed false lumen. Anticoagulation with heparin was initiated to prevent further thrombus formation and occlusion, which alleviated the patient’s symptoms. Anticoagulant therapy with aspirin was continued at the outpatient clinic. Chronic-phase assessments showed well-maintained SMA perfusion (**Fig. 3**).

As a result, 13 patients received conservative treatment, while only 1 patient underwent extra-anatomical bypass. All patients survived without complications, including intestinal necrosis, throughout the long-term follow-up period. The mean duration of follow-up was 7.20 ± 3.21 years, during which all patients remained healthy with no recurrence of abdominal pain. The false lumen disappeared in 3 patients between 6 and 36 months of age, and only 1 patient exhibited an enlargement of the false lumen. Heparinization has proven to be a sufficient treatment option for maintaining intestinal perfusion during the acute phase or at disease onset. For long-term management, aspirin was administered to 9 patients, and a direct oral anticoagulant (DOAC) was administered to 1 patient, which could be discontinued if no stenosis of the SMA trunk was confirmed by follow-up CTA.

## Discussion

SIDSMA can cause life-threatening complications. A recent review of this pathological condition reported a bowel resection rate of 2.1%, which was correlated with mortality rates.^2)^ Over 90% of patients can be managed with conservative treatment. In our series, only 1 patient required an extra-anatomical autologous graft bypass due to intestinal ischemia from SMA occlusion, which successfully prevented intestinal necrosis. Similarly, Mizuno et al.^7)^ reported 221 patients, including 12 who underwent surgical procedures, with 4 patients requiring extra-anatomical bypass surgery. Satokawa et al.^8)^ documented 30 patients, 2 patients with extra-anatomical bypass surgery and 1 patient with endovascular surgery. Notably, as these reports suggest, most cases originate from East Asian countries such as Japan, South Korea, and China.^2)^ With advancements in diagnostic imaging, particularly CT, the detection rate of such cases is expected to increase. Furthermore, a potential association between COVID-19 and this condition has been suggested,^9)^ indicating a possible global increase in cases. It is vital to evaluate both acute-phase outcomes and long-term results to refine treatment strategies. Endovascular stenting (EST) for SIDSMA is gaining popularity.^5)^ The European Society for Vascular Surgery guidelines recommend considering EST in patients with symptomatic SIDSMA who do not respond to medical treatment and are suspected of having bowel ischemia (Class IIa, Level C).^10)^

**Fig. 4:**
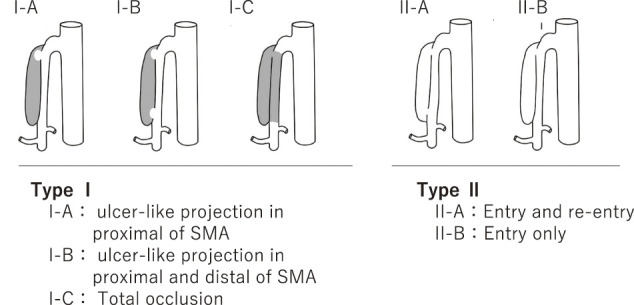
We propose dividing Sakamoto’s classification^1)^ of SIDSMA into 2 groups. Type I: With thrombosed false lumen, which may cause true lumen stenosis due to thrombosis in the false lumen. Type I-A: Ulcer-like projection in proximal SMA. Type I-C: Total occlusion. Type I-B: Ulcer-like projection in proximal and distal SMA. Type II: Without thrombosis in the false lumen. Type II-A: Entry and re-entry. Type II-B: Entry only without stenosis. SMA: superior mesenteric artery

Hypertension, smoking, and arteriosclerosis are risk factors.^11)^ Additionally, a large aortomesenteric angle is considered a contributory factor to SIDSMA,^2)^ potentially causing mechanical stress and high blood pressure, which are significant risk factors for this condition. Emergency CTA plays a crucial role in diagnosing SIDSMA and evaluating whether secondary intestinal ischemic lesions requiring bowel resection are present.^2)^ Yun et al.^12)^ added complete occlusion of the SMA to the Sakamoto’s classification^1)^ of SIDSMA. Zerbib et al.^13)^ modified Sakamoto’s classification into 6 categories. We considered the critical factor to be whether the bowels were at risk of ischemia. The pathology influencing our treatment decisions was true luminal patency along with stenosis and thrombosis formation in the false lumen, which is crucial for deciding how to treat patients with SIDSMA in the emergency room. In our series, based on Sakamoto’s classification of SIDSMA, no bowel ischemia was confirmed, so conservative treatment was performed for these patients, yielding sufficient long-term outcomes. Our proposed simplified classification (Yu-Kondo’s classification; **Fig. 4**) is intended to guide acute-phase decision-making. Patients with thrombosed false lumens (Type I) should be carefully evaluated for signs of intestinal ischemia, as these lesions are more likely to compress the true lumen and compromise blood flow. However, we could not provide enough data to confirm the validity of this classification with long-term results in this article. We also think that the morphology of the arterial dissection may affect subsequent aneurysm formation and alterations in blood flow. A morphology-based classification is necessary to explore these relationships in our further research.

Satokawa et al. reported that morphological changes in SIDSMA primarily occur within the first year after disease onset, based on a follow-up of 170–799 days.^14)^ However, long-term follow-ups have seldom been reported, and large dissecting aneurysms can be detected at various time points using CTA.^2)^ If the state of the dissection changes over the long term, there is a possibility that, similar to preemptive TEVAR in Stanford type B acute aortic dissection, the treatment strategy for SIDSMA may evolve based on long-term outcomes. In analogy to the TEVAR strategy for uncomplicated type B aortic dissection, where anatomical progression such as false lumen enlargement or sac formation prompts intervention, similar morphological surveillance in SIDSMA could potentially inform future indications for endovascular therapy. We hypothesized that morphological changes in the false lumen of SIDSMA might resemble those seen in type B aortic dissection. Among the known positive predictors of false lumen enlargement in type B dissection, we focused on 3 features that are assessable by Yun’s classification^12)^—patency of the false lumen, presence of a single entry, and partial thrombosis of the false lumen—evaluated them during follow-up CT imaging. In our series, conservative treatment was administered to 13 patients, yielding satisfactory long-term outcomes over 7 years without the need for additional interventions. Although morphological progression, enlargement of the false lumen, was observed in 1 patient during long-term follow-up, no clinical symptoms or need for reintervention were noted in these cases. These findings suggest that radiological changes alone may not necessitate therapeutic intervention. This highlights the importance of correlating imaging findings with clinical status when managing SIDSMA patients over time. To date, there are no data suggesting that surgical intervention during the acute phase improves long-term prognosis (**Table 1**). Our findings support the continuation of conservative treatment for patients with SIDSMA. Managing blood pressure is crucial for patients with SIDSMA, not only during the acute phase but also throughout the chronic phase, as it helps prevent enlargement of the false lumen or aneurysm formation. In several cases, heparin was administered to maintain intestinal perfusion and to prevent further thrombus formation during the acute phase or at the onset of dissection. Aspirin-based antiplatelet therapy was given to 9 outpatients at a high risk of thrombotic or embolic events. However, this treatment strategy may require reconsideration, as SIDSMA is not an atherosclerotic disease,^15)^ and there is no evidence supporting the beneficial effect of antithrombotic therapy.^16)^ A recent meta-analysis did not recommend the use of additional antithrombotic agents for either symptomatic or asymptomatic SMA dissection, providing no further evidence of beneficial effects.^17)^

## Conclusion

Long-term outcomes of conservative treatment for patients with SIDSMA are satisfactory. To achieve these results, 2 key principles are crucial: intensive conservative management and timely surgical intervention in patients exhibiting signs of intestinal ischemia during the acute phase. Continued close observation is necessary to establish optimal treatment strategies for SIDSMA.
